# Response Letter to the Editor in Reference to Letter to the Editor about “Does Overall Catastrophic Health Care Expenditure Make Sense”?

**Published:** 2018-08

**Authors:** Manal ETEMADI, Aidin ARYANKHESAL

**Affiliations:** Dept. of Health Services Management, School of Health Management and Information Sciences, Iran University of Medical Sciences, Tehran, Iran

## Dear Editor-in-Chief

We note the author(s) of the letter entitled “Does ‘overall catastrophic health care expenditure’ make sense?” Meta-analyses often include studies that are different from each other in important ways; hence heterogeneity may also be due to differences in study design or patient characteristics across studies (1). Heterogeneity is usually expected in meta-analyses and it would be surprising if multiple studies, performed by different teams in different places with different methods, all end up with similar estimation of the underlying parameter (2).

Heterogeneity is one of limitations that researchers deal with when combining the results of individual studies, while it could be our greatest ally when we explore its sources (3). Heterogeneity of study results can lead to helpful insights into the problems being addressed. An important general principle is that there were strong a priori reasons for choosing the particular factors by which the authors stratified their findings (4), as we carried out in this study. Therefore, heterogeneity of study results can provide many benefits and should be viewed as strength of meta-analysis, not a barrier to its use (4).

Significant statistical heterogeneity arising from methodological diversity or differences in outcome assessments suggests that the studies are not all estimating the same quantity. However, the heterogeneity does not necessarily suggest that the true intervention effect or the analyzed Index varies. In particular, heterogeneity associated solely with methodological diversity would indicate the studies suffer from different degrees of bias (5).

In our study (6), heterogeneity was expected because most studies estimated Catastrophic Health Expenditure (CHE) for their local province, which naturally can differ from that of other provinces or regions of Iran due to specific socio-economic status of any province. Carrying out subgroup analyses or stratification was one important way to identify the main sources of heterogeneity among studies (7). Hence we performed subgroup meta-analyses to assess the influence of the study area (community, hospital, rural and urban) on the result. Therefore, we addressed one aspect of heterogeneity in our study design in sub-group analysis. In fact, we identified sources of heterogeneity in advance of the analysis that helps to be interpreted more easily (4). In addition, using PICO format and developing explicit list of inclusion and exclusion criteria and identifying a priori hypothesis to explain anticipated heterogeneity (8) helped us to reduce heterogeneity. At the end of the results we also developed a longitudinal analysis to demonstrate the trend of changes in the CHE, which also give some explanations to the heterogeneity, because as the graph shows, the CHE had some fluctuations.

Nevertheless, meta-analysis of sensitive issues such as catastrophic health expenditure should cite with caution due to the possible bias in primary research results and presence of possible heterogeneity sources.
